# Impact of Hemodialysis on Dyspnea and Lung Function in End Stage Kidney Disease Patients

**DOI:** 10.1155/2014/212751

**Published:** 2014-05-08

**Authors:** Anastasios F. Palamidas, Sofia-Antiopi Gennimata, Foteini Karakontaki, Georgios Kaltsakas, Ioannis Papantoniou, Antonia Koutsoukou, Joseph Milic-Emili, Demetrios V. Vlahakos, Nikolaos G. Koulouris

**Affiliations:** ^1^Respiratory Function Laboratory, 1st Respiratory Medicine Department, Athens University Medical School, “Sotiria” Hospital, 152 Mesogeion Avenue, Athens 11527, Greece; ^2^Artificial Kidney Unit, Euromedica Athinaion, Kononos Street 121-123, Vyronas, Attica, 16231 Athens, Greece; ^3^Meakins-Christie Labs, McGill University, 3626 Saint Urbain Street, Montreal, QC, Canada; ^4^Renal Unit, Attikon University Hospital, Rimini 1, Haidari, Attika, 12462 Athens, Greece

## Abstract

*Background*. Respiratory symptoms are usually underestimated in patients with chronic kidney disease undergoing maintenance hemodialysis. Therefore, we set out to investigate the prevalence of patients chronic dyspnea and the relationship of the symptom to lung function indices. * Methods*. Twenty-five clinically stable hemodialysis patients were included. The mMRC dyspnea scale was applied before and after hemodialysis. Spirometry, single breath nitrogen test, arterial blood gases, static maximum inspiratory (*P*_*i*max⁡_) and expiratory (*P*_*e*max⁡_) muscle pressures, and mouth occlusion pressure (*P*_0.1_) were also measured. * Results*. Despite normal spirometry, all patients (100%) reported mild to moderate degree of chronic dyspnea pre which was reduced after hemodialysis. The sole predictor of (Δ) mMRC was the (Δ) *P*_0.1_ (*r* = 0.71, *P* < 0.001). The *P*_*i*max⁡_ was reduced before and correlated with the duration of hemodialysis (*r* = 0.614, *P* < 0.001), whilst after the session it was significantly increased (*P* < 0.001). Finally (Δ) weight was correlated with the (Δ) *P*_*i*max⁡_  %pred (*r* = 0.533, *P* = 0,006) and with the (Δ) CV (%pred) (*r* = 0.65, *P* < 0.001). * Conclusion*. We conclude that dyspnea is the major symptom among the CKD patients that improves after hemodialysis. The neuromechanical dissociation observed probably is one of the major pathophysiologic mechanisms of dyspnea.

## 1. Introduction

Renal injury is not only a localized disease, eventually leading to a progressive and irreversible loss of nephron mass and functions, but also a syndrome affecting multiple organ systems. Among those, CKD and KRT options, such as hemodialysis and peritoneal dialysis, severely affect the respiratory system. Specifically, their effects are (a) acute: causing infections, pleural effusions, and ARDS and (b) chronic: leading to calcification of the lung parenchyma and finally respiratory impairment [1].

Despite the increasing interest in lung pathology during ESKD and the complications that may arise from the maintenance therapeutic options, respiratory symptoms are usually either underestimated or overlooked. The physical symptom burden though is the primary parameter defining the quality of life of CKD patients [2]. Furthermore, hemodialysis maintenance therapy seems to cause a definite regression of these symptoms. Specifically, symptoms concerning the respiratory system are not well documented [2, 3]. One of the commonest respiratory symptoms of ESKD is dyspnea [4], usually assessed with lung function testing. Several researchers explored CKD effects, the impact of hemodialysis on lung function, and dyspnea with ambiguous results. Anemia and gas transfer defects [5], fluid overload and premature airway closure [6], ventilation-perfusion mismatching [6], hypoxemia due to centrally driven hypoventilation [7], uremic respiratory muscle dysfunction, and severe mechanical loading [8] were reported in these patients. Several functional pulmonary abnormalities, including restriction, obstruction [9, 10], and impaired diffusion capacity [11], have also been described. Surprisingly, scarce reports exist for measuring, explaining, and categorizing chronic dyspnea in relation to lung function indices and the way the CKD and KRT affect it in these patients.

Thus we set out to investigate the following: (a) the prevalence, (b) the degree, and (c) the relationship of chronic dyspnea with lung function parameters, both before and after hemodialysis. Therefore, we studied the prevalence and the degree of chronic dyspnea in ESKD patients before and after hemodialysis and we wondered whether it is correlated with any of the commonly used lung function indices.

## 2. Materials and Methods

### 2.1. Subjects

The study population consisted of twenty-five (25) ambulatory Caucasian patients (15 men) with ESKD (GFR < 10 mL min^−1^) [12] undergoing maintenance hemodialysis every other day. These patients were candidates for renal transplantation and were referred to our laboratory for preoperative respiratory function assessment. Thus, this population was highly selected and neither inflammation nor malnutrition was observed. The causes of ESKD were diabetes mellitus type II (40%), arterial hypertension (20%), glomerulonephritis (20%), and cystic kidney disease (20%). Their anthropometric characteristics, blood serum, and lung function data are shown in Tables 1 and 2.

Inclusion criteria were (a) age above 18 years, (b) ability to perform full lung function testing satisfactorily, and (c) stable clinical and functional state for at least four weeks before testing. Exclusion criteria were (a) cardiovascular disorders, ruled out by a cardiologist, (b) known lung disease, such as asthma or COPD, (c) neuromuscular disorders, (d) previous thoracic or abdominal surgery, (e) pleural disease, and (f) malnutrition. Twelve out of 25 patients have never been smokers and no patient was cachectic (BMI < 18).

The study had the approval of the hospital's ethics committee. All subjects gave informed consent.

### 2.2. Hemodialysis

All patients included in the study group suffered from established CKD according to the KOQI [12] with a predicted GFR < 10 mL min^−1^. They were receiving hemodialysis three times a week as maintenance therapy. This was applied via a forearm arteriovenous fistula with extracorporeal blood flow rates between 250 and 350 mL min^−1^ using a dialyzer (AK 200 Gambro, Stockholm, Sweden). Bicarbonate buffer with high concentration of HCO_3_ (35 mmol/L) and disposable synthetic biocompatible dialyzer membranes (polysulfone hollow fiber dialyzer membranes, Toray TS series, Japan) were used in all sessions. The duration of hemodialysis, the patients weight, and blood serum data are given in Table 1.

### 2.3. Chronic Dyspnea and Lung Function Testing

Chronic dyspnea was rated according to the mMRC 6-point scale [13]. Simple spirometry was measured with the *V*_max⁡_ apparatus (*V*_max⁡_ Encore 22: Sensor Medics, Yorba Linda, CA, USA) using the “fast inspiratory maneuver” [14]. Static lung volumes were measured with the multiple nitrogen washout technique [15] (*V*_max⁡_ Encore 22, Sensor Medics). The DL_CO_ single breath technique was also determined (*V*_max⁡_ Encore 22, Sensor Medics) [16]. Predicted values for spirometry, static lung volumes, and DL_CO_ were from the European Community for Coal and Steel [17]. The arterial pH and arterial partial pressures of oxygen (PaO_2_) and carbon dioxide (PaCO_2_) (mmHg) were measured with the Stat Profile Critical Care Xpress apparatus (Nova Biomedical, Waltham, MA, USA).

The SBN_2_ was also performed with the *V*_max⁡_ apparatus (*V*_max⁡_ Encore 22, Sensor Medics). Subjects were asked to exhale to RV, and after an inhalation of 100% oxygen to TLC, they were asked to slowly exhale (≤0.5 L/s) to RV again. The SBN_2_ technique was used to obtain the CC, the CV, and the slope Δ*N*_2_/Δ*V*.

Static *P*_*i*max⁡_ and *P*_*e*max⁡_ were measured with a plastic semirigid flanged mouthpiece fitted to a metallic stem incorporating a 3-way tap manufactured according to the design of Ringqvist [18]. A differential pressure transducer (Validyne MP45-36-871, Validyne Co., Northridge, CA) with a range of ±340 cm H_2_O was connected to the 3-way tap with a 70 cm fine polyethylene catheter. The pressure transducer was calibrated before each study using a U-tube water manometer. Pressures signals were displayed on a computer screen. The subjects used their hands to hold the lips firmly onto the mouthpiece if a leak was noticed. Prior to a *P*_*e*max⁡_ or *P*_*i*max⁡_ effort, the operator closed the 3-way tap with the subject at TLC or RV, respectively. All subjects were given verbal encouragement. A period of learning the procedure preceded the definitive measurements [18, 19]. All measurements followed the criteria of Ringqvist [18] such that (a) no extra leakage occurred, (b) the three highest pressures were similar (within 5%) and later attempts did not yield higher results, and (c) the subjects felt that they had given a maximum effort. At least 1 min rest was allowed between efforts. Pressures maintained for less than 1 second were disregarded. The highest pressures generated by an individual were used for analysis. Predicted values for *P*_*e*max⁡_ and *P*_*i*max⁡_ standardized for age, height, and gender were obtained from Wilson et al. [19].

Pattern of breathing (*V*_*E*_, *V*_*T*_, *T*_*E*_, *T*_*I*_, *T*_TOT_, RR, *V*_*T*_/*T*_*I*_, *T*_*I*_/*T*_TOT_) was also assessed during resting breathing. Subjects with a nose clip on breathed through a heated pneumotachograph (Screenmate-Box, Erich Jaeger GmbH & Co., Germany) connected to a differential pressure transducer. Tidal volume was measured by integrating the flow signal. Inspiratory time and total breath cycle duration were also measured from the flow signal. In order to minimize the effects of anxiety, all indices were measured after the patient had become familiar with the procedure and actual measurements were made only when ventilation had remained constant for at least ten minutes [20]. Normal values for the pattern of breathing were obtained from Tobin et al. [21].

Respiratory drive as reflected in *P*_0.1_ measurement was defined as the pressure change during the first 100 msec from the onset of airflow of a patient's spontaneously initiated breath [22]. It was measured using a three-way helium valve (Hans—Rudolph, Kansas City, MO, USA). The latter was occluded randomly at the end of expiration by a helium pneumatic valve. The valve was connected to a heated pneumotachograph (Screenmate-Box, Erich Jaeger GmbH & Co., Germany) while patients were seated comfortably in a quiet room with a nose clip on. A learning period preceded the definitive measurements. To eliminate stress and any other external influences, patients wore earplugs to prevent any acoustic stimuli while the operator was standing behind the patient in order to avoid any optic interference. Patients were unable to anticipate occlusions, which were applied silently and randomly according to the technique of Whitelaw et al. [23]. Normal values were obtained from the ATS/ERS statement on respiratory muscle testing [24]. At least 5 measurements were obtained for each subject. The mean value of the 5 best efforts was used for analysis.

### 2.4. Procedure

Patients had undergone a CXR, biochemistry, and a cardiovascular assessment obtained no more than 7 days before the examination day. On the day of examination each of the 25 patients signed an informed consent form. Then medical history was recorded and full physical examination, weighing of the patient, and arterial blood gases were performed; these were followed by full lung function testing (spirometry, static lung volumes, DL_CO_, SBN_2_, *P*_*i*max⁡_ and *P*_*e*max⁡_ pattern of breathing, and *P*_0.1_) before and immediately after hemodialysis maintenance.

### 2.5. Statistical Analysis

Data are expressed as mean ± (SD), unless otherwise stated. For comparisons paired *t* test or Wilcoxon's nonparametric test for paired data was used where appropriate. Correlations between mMRC dyspnea scale and various variables were calculated using Spearman's correlation coefficient. Multiple linear regression and backward stepwise regression analysis were used to identify the significant variables. A *P* ≤ 0.05 value was considered as significant. Statistical analysis was performed using SigmaStat (V3.5) and SigmaPlot (V10.0) (Jandel Scientific, CA, USA) statistical software.

## 3. Results

Anthropometric, hemodialysis, and blood serum data are presented in Table 1. We included smokers (*n* = 13, pack years 31 ± 23), who were advised to abstain from their habit for at least 24 hours before performing full lung function testing, and nonsmokers (*n* = 12). Electrolytic disturbances were noted, namely, hypercalcemia (adjusted to the albumin levels) and hyperkalemia.

**Figure 1:**
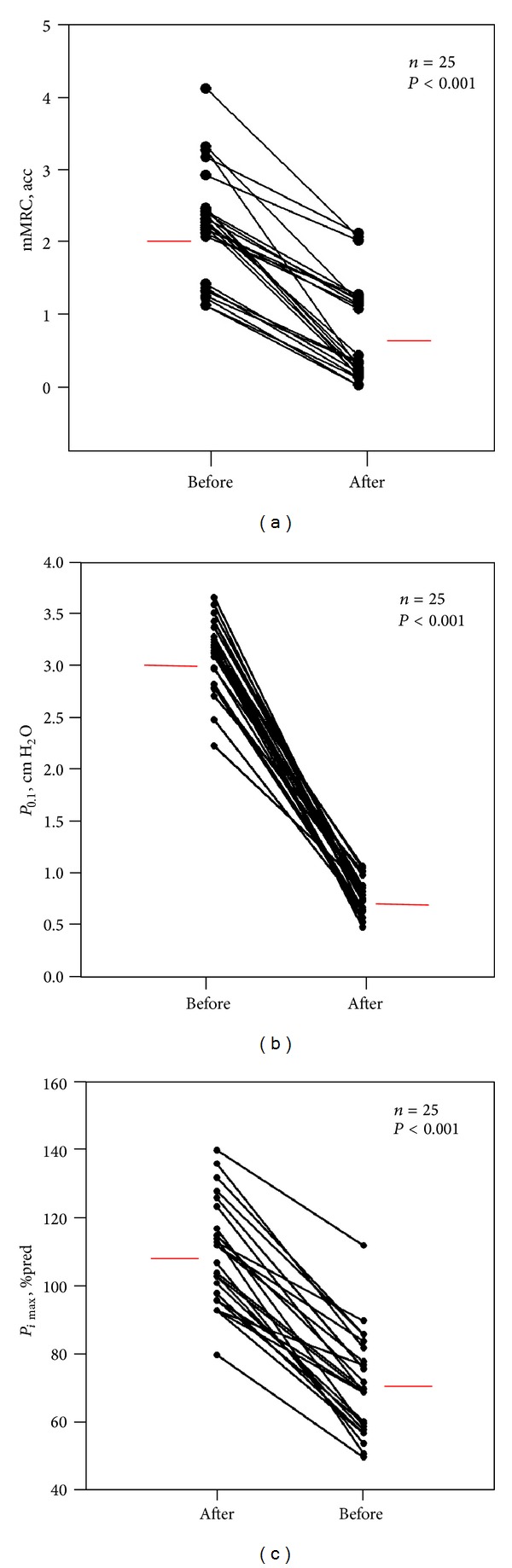
(a) mMRC, (b) *P*_0.1_—ventilatory drive, and (c) *P*_*i*max⁡_ before and after hemodialysis are shown. Black dots represent the individual patients and red lines represent the mean values before and after hemodialysis.

Body weight was reduced significantly by 3 Kgs (*P* < 0.001, 74 ± 12 before to 71 ± 12 after dialysis) (Table 2). The mMRC 6-point scale was also significantly reduced (Figure 1(a)). Routine lung function indices were within normal limits and they did not change significantly after hemodialysis.

**Figure 2:**
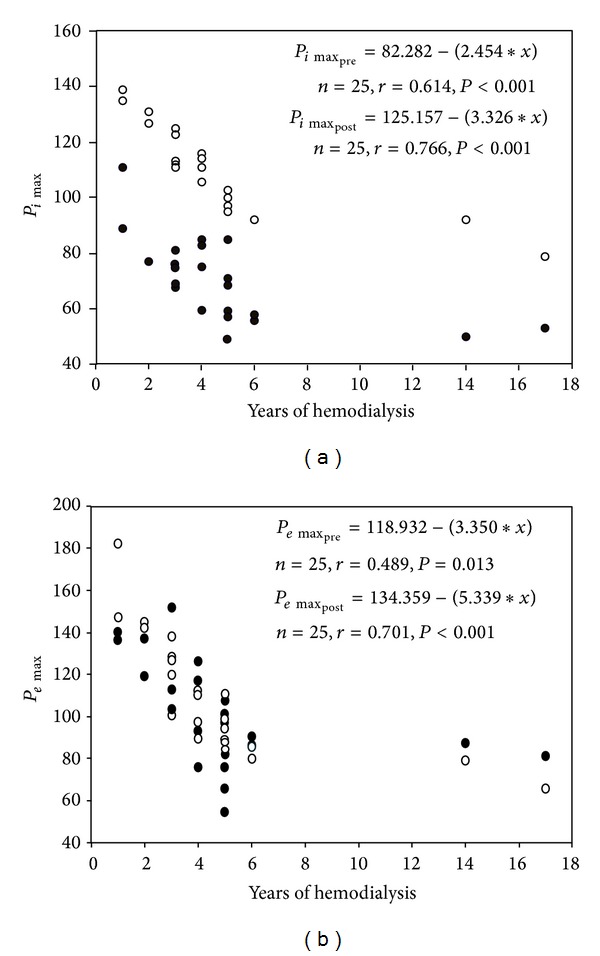
(a) Relationship of the *P*_*i*max⁡_ with the duration of hemodialysis for the 25 patients before and after hemodialysis (*r* = 0.614, *P* < 0.001 and *r* = 0.766, *P* < 0.001, resp.). Black dots: *P*_*i*max⁡_ before hemodialysis. White dots: *P*_*i*max⁡_ after hemodialysis. (b) Relationship of the *P*_*e*max⁡_ with the duration of hemodialysis for the 25 patients before and after hemodialysis (*r* = 0.489, *P* = 0.013 and *r* = 0.701, *P* < 0.001, resp.). Black dots: *P*_*e*max⁡_ before hemodialysis. White dots: *P*_*e*max⁡_ after hemodialysis.

An analysis of the SBN_2_ technique performed (23 subjects) is shown at Table 2 (in 2 patients CC and CV could not be calculated). The Δ*N*_2_/Δ*V* and CV were increased before hemodialysis but were significantly reduced returning to normal limits after hemodialysis in all but 3 patients. The CC was increased before hemodialysis, but was also significant decreased after the hemodialysis session. There were no significant differences between groups, when SBN_2_ data were tested separately for smokers and nonsmokers. The *P*_*i*max⁡_ before and after hemodialysis (*r* = 0.614, *P* < 0.001 and *r* = 0.766, *P* < 0.001, resp.) was well correlated with the duration of hemodialysis maintenance therapy whilst the same result was observed for the *P*_*e*max⁡_ before and after the session (*r* = 0.489, *P* = 0.013 and *r* = 0.701, *P* < 0.001, resp.) (Figures 2(a) and 2(b)). Additionally *P*_*i*max⁡_ was reduced significantly (Figure 1(c)), whilst *P*_*e*max⁡_ remained unchanged after hemodialysis (Table 2).

Pattern of breathing and arterial blood gases data are shown in Table 3. All patients were hyperventilating as indicated by the high *V*_*E*_ and RR before and after hemodialysis. The *P*_0.1_ was increased before hemodialysis and decreased significantly after hemodialysis in all patients (Figure 1(b)). The *P*_0.1_/*V*_*T*_ % pred VC ratio was also significantly reduced. The pH became alkalotic whilst PaCO_2_ tension was significantly increased but remained below the normal limits. The PaO_2_ was decreased after the session but no hypoxemia was observed.

**Figure 3:**
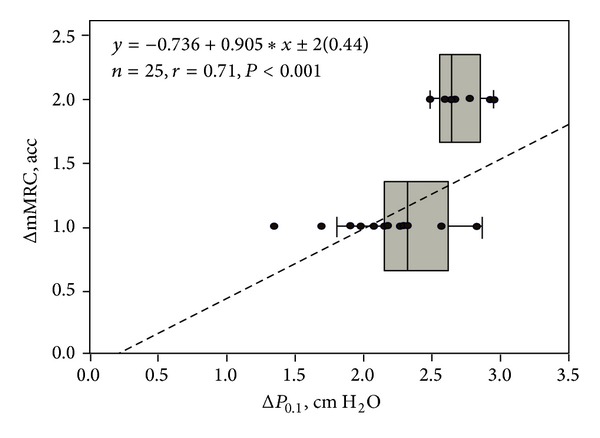
Box plot showing the relationship of (Δ) mMRC with (Δ) *P*_0.1_ before and after hemodialysis. Dashed line: regression line. Solid lines: median lines of the change (Δ) of *P*_0.1_ at ΔmMRC values of 1 and 2, respectively. Regression equation: *y* = −0.736 + (0.905∗*x*) ± 2(0.44), *n* = 25, *r* = 0.71, and *P* < 0.001. The slope of the line indicates that the (Δ) mMRC decreases, on average, by one unit per 0.9 units decrease of the (Δ) *P*_0.1_.

**Figure 4:**
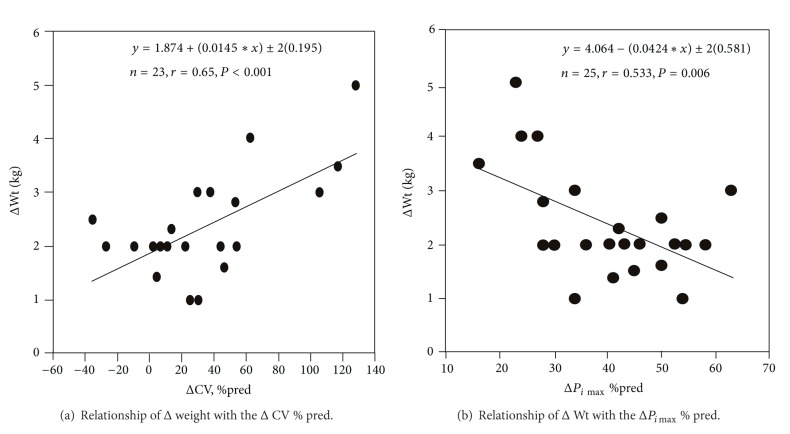
(a) Relationship of (Δ) Wt with the (Δ) CV % pred. Solid line: regression line. Regression equation: *y* = 1.874 − 0.0145∗*x* ± 2(0.195) ,   *n* = 23, *r* = 0.65, *P* < 0.001. Subjects, represented as black dots, are 23 because closing volume could not be calculated in 2 patients. (b) Relationship of the (Δ) Wt to (Δ) *P*_*i*max⁡_ % pred. Solid line: regression line. Regression equation: *y* = 4.064 − (0.0424∗*x*) ± 2(0.581), *n* = 25, *r* = 0.533, and *P* = 0.006. The 25 subjects are represented as black dots.

The (Δ) mMRC was correlated with the (Δ) *P*_0.1_ (*P* < 0.001) before and after dialysis. Multiple regression analysis showed that the sole predictor of (Δ) mMRC grade was the (Δ) *P*_0.1_ (*r* = 0.71, *P* < 0.001) (Figure 3). Furthermore, the observed (Δ) Wt was correlated with the (Δ) *P*_*i*max⁡_ % pred. (*r* = 0.533, *P* = 0,006) and (Δ) CV (% pred.) (*P* = 0.0008) (Figures 4(a) and 4(b)). Backward stepwise regression revealed that (Δ) CV (% pred.) was proportionally reduced to (Δ) weight (*r* = 0.65, *P* < 0.001). Finally, a weak correlation was detected between the (Δ) *V*_*E*_ and the (Δ) pH (*r* = 0.44, *P* = 0.03) before and after hemodialysis.

## 4. Discussion

The main findings of the present study in patients with ESKD undergoing hemodialysis maintenance therapy, awaiting kidney transplantation, are the following. (1) All patients (100%) reported some degree of chronic dyspnea before hemodialysis maintenance, which was reduced significantly after the treatment. (2) *P*_0.1_ was significantly reduced after hemodialysis. (3) *P*_*i*max⁡_ was reduced before and significantly increased after hemodialysis, whilst *P*_*e*max⁡_ remained unchanged.

### 4.1. Chronic Dyspnea

Scarce reports exist on the prevalence and grading of chronic dyspnea in ESKD patients receiving hemodialysis maintenance therapy. Dyspnea prevalence has not been adequately studied and the main reason is that no author has applied a widely accepted and validated tool such as the mMRC scale. Furthermore, the (Δ) mMRC has not been reported immediately after the hemodialysis maintenance sessions. Therefore dyspnea is either underestimated or overlooked. The reported prevalence of dyspnea was 20 to 60% [2–4]. In the present study, all patients (100%) reported mild to moderate degree of chronic dyspnea before and exhibited a significant reduction after hemodialysis (Figures 1(a) and 3). Dyspnea improvement was not correlated with any of the changes in the arterial blood gases. Finally after hemodialysis in half of the patients (13/25), the sensation of dyspnea was abolished.

### 4.2. Pattern of Breathing

To the best of our knowledge there are a few studies examining *P*_0.1_ before and immediately after hemodialysis maintenance therapy [25, 26] in ESKD patients. In spontaneous breathing patients Sebert et al. [26] examined the patient's *P*_0.1_ relationship with the *V*_*E*_ at different CO_2_ concentrations, before and after hemodialysis maintenance. They found that for the same *V*_*E*_ response CKD patients needed a higher neuromuscular ventilatory drive than normal subjects. The latter response was not corrected after the hemodialysis maintenance session. They attributed their results to a neuromuscular hypoexcitability that did not respond to hemodialysis therapy. Only one other study, that of Huang et al. [25], examined the effect of hemodialysis on *P*_0.1_ in mechanically ventilated patients. They reported that *P*_0.1_ was reduced after hemodialysis. Their finding was attributed to the reduced work of breathing and to the lung mechanics improvement after the hemodialysis session. In our spontaneously breathing patients *P*_0.1_ was increased before but significantly decreased after hemodialysis (Table 3) (Figure 1(b)). To the best of our knowledge there is no report measuring *P*_0.1_ before and after maintenance hemodialysis in spontaneously breathing patients. A significant correlation was detected between (Δ) mMRC grade and (Δ) *P*_0.1_ (*P* < 0.001) (Figure 3) before and after hemodialysis. Multiple regression analysis (*r* = 0.71, *P* < 0.001) revealed that the sole predictor of the (Δ) mMRC chronic dyspnea magnitude was the change in the ventilatory drive assessed by the (Δ) *P*_0.1_. Specifically the slope of the regression indicated that, for 1 unit reduction of mMRC after hemodialysis, the *P*_0.1_ is changing also by approximately one unit (0.9). Additionally we observed a paired pattern of the (Δ) mMRC scale and the (Δ) *P*_0.1_ after hemodialysis. Thus the patients with the higher (Δ) mMRC chronic dyspnea scale exhibited the largest (Δ) *P*_0.1_ values.

After the hemodialysis maintenance the significant drop in *P*_0.1_ can be attributed to many factors. (a) Better neuromechanical coupling: the *P*_0.1_/*V*_*T*_ % VC was significantly decreased after the hemodialysis maintenance therapy session (Table 3), reflecting a better neuromuscular coupling. Unfortunately this ratio was not correlated with the dyspnea scale change. (b) Depression of the ventilatory drive and profound hypoventilation because of the alkalization of body fluids after bicarbonate hemodialysis: hyperventilation due to the metabolic acidosis occurring during the interdialytic period is well documented in patients with ESKD on hemodialysis maintenance therapy [27]. After hemodialysis, breathing pattern irregularities and hypoventilation hypoxemia are frequently observed, due to the transient alkalization of the internal milieu and CO_2_ unloading into the dialytic regiment, as De Backer et al. noted [27, 28]. In the present study, using biocompatible membrane and bicarbonate enriched dialytic regiments, applied uniformly to all patients and thus preventing significant CO_2_ unloading, the pH became alkalotic as a result of the PaCO_2_ and HCO_3_ significant increase (Table 3). Despite the latter, patients still exhibited hyperventilation, albeit the small tendency to decrease after the hemodialysis session, as *V*_*E*_ remained practically unchanged (Table 3). Furthermore, we did not note any hypoxemia nor any correlations of the *P*_0.1_ with the pH or with any other blood gas exchange indices, in consistency with Herrera et al. [29]. Only a weak correlation was detected between the (Δ) *V*_*E*_ and the (Δ) pH (*r* = 0.44, *P* = 0.03) before and after hemodialysis. The reason of the increased peripheral chemosensitivity after hemodialysis still remains controversial, and it is still under investigation. The disequilibrium syndrome observed in the uremic patients due to the abrupt changes of the blood internal milieu is proposed as a potential cause in the ventilatory responses of the central nervous system in ESKD patients on hemodialysis maintenance therapy [30]. Finally, it is not clear if the reduction of dyspnea resulted from the improvement of the ventilatory drive or* vice versa*. Further studies are needed to elucidate these findings.

### 4.3. Respiratory Muscles

Another explanation for the significant drop in *P*_0.1_ immediately after hemodialysis in ESKD patients may arise from the suggested mechanical unloading of the respiratory muscles and the overall improvement of chest mechanics after the hemodialysis session. Skeletal muscle dysfunction is a well-described entity of uremia. Uremic myopathy [31] can be attributed to (a) structural changes, such as a decrease in the proportion of contractile tissue to collagen fibers in the sarcomere, resulting from malnutrition, uremic toxins, and acidosis, and (b) inefficient contractility because of hypercalcemia and secondary hyperparathyroidism. Several studies have investigated the respiratory muscle function of patients with ESKD [31–35] but with controversial results. On one hand, Bark et al. [31] suggested that the excitability-contractility coupling of respiratory muscles is deranged, resulting from the aluminum compounds frequently used in the hemodialysis regiments. The observed hypoexcitability was fully reversed in their cohort after the hemodialysis session. On the contrary, Karacan et al. [35] described a pronounced decrease in the inspiratory and expiratory muscle strength that was not reversed after a hemodialysis session. Our data show that *P*_*i*max⁡_ is compromised, whilst *P*_*e*max⁡_ is generally preserved. The *P*_*i*max⁡_ decreased (70 ± 14 cm H_2_O) in all patients before the hemodialysis (Figure 1(c)), whilst *P*_*e*max⁡_ was within normal limits (103 ± 24 cm H_2_O). After the hemodialysis session though all patients (Figure 1) exhibited a significant increase in *P*_*i*max⁡_ values (109 ± 15 cm H_2_O). Our findings are in agreement with the Kovelis et al. [32] study which showed that the reduction of the *P*_*i*max⁡_ is correlated with the duration of hemodialysis before and after the session (*r* = 0.614, *P* < 0.001 and *r* = 0.766, *P* < 0.001, resp.), whilst very few patients exhibited expiratory muscle weakness (Figure 2). The *P*_*e*max⁡_ was preserved, although a small tendency to decrease with the duration of dialysis was observed before and after the session (*r* = 0.489, *P* = 0.013 and *r* = 0.701, *P* < 0.001, resp.) (Table 3) (Figure 2). The surprising finding is that our data show a significant increase towards normal of the *P*_*i*max⁡_ after a single dialysis session whilst the *P*_*e*max⁡_ remained unchanged although it tended to increase (Table 3). A possible explanation is the fact that CKD affects selectively only the large nerve fibers such as the phrenic nerve which innervates the largest inspiratory muscle (the diaphragm), whilst the nerves innervating the expiratory muscles are not of large caliber nerves [36, 37]. In the preliminary study of Zifko et al. [34] delayed phrenic nerve latencies are observed in patients with CKD on maintenance hemodialysis, suggesting that phrenic neuropathy is an observed complication of uremia. Corroborative evidence of this selectivity is the fact that our group of patients had reduced *P*_*i*max⁡_, but very few patients had reduced *P*_*e*max⁡_. These patients with reduced *P*_*e*max⁡_ had also reduced *P*_*i*max⁡_.

Additionally the (Δ) *P*_*i*max⁡_ % pred. was correlated with the (Δ) weight (*r* = 0.533, *P* = 0,006) (Figure 4(b)). The (Δ) weight was also correlated, but stronger, with the (Δ) CV (*r* = 0.65, *P* < 0.001). Backward stepwise regression analysis revealed that the sole predictor of the (Δ) CV was the (Δ) weight (Figure 4(a)), in accordance with the study of Zidulka et al. [6]. We assume that premature airway closure and gas trapping occur during the interdialytic period because of the accumulation of excess lung water and subclinical pulmonary edema. The latter increases the work of breathing and the load of the already impaired muscles by the uremic neuropathy and myopathy. Thus, the neuromuscular dissociation becomes apparent before the hemodialysis maintenance therapy. As a result a higher ventilatory drive (*P*_0.1_) reflecting an augmented respiratory effort was necessary to overcome the disadvantaged mechanical load and the neuromechanical dissociation. This neuromechanical dissociation can be perceived as dyspnea from the patients [38]. The limitation of this theory is that the (Δ) weight is not a reliable marker of the total lung water as Wallin et al. [5] reported. Unfortunately, in the present study we did not measure the total lung water removed during hemodialysis. After the dialysis session the neuromechanical coupling is improved because of the unloading of the muscles expressed as the significant increase in *P*_*i*max⁡_ and the improvement of neuropathy because of the ultrafiltration of the middle sized molecules and the restoration of the electrolytic imbalances [37].

### 4.4. Clinical Implications

The disease symptoms burden affects therapy compliance and hence the therapeutic outcome [2–4]. Whether the removal of excess lung water with aggressive ultrafiltration could imply greater reduction of patient's chronic dyspnea and thus better compliance needs to be further explored.

Respiratory muscles dysfunction leads to breathlessness and ventilatory failure [39]. It should be noted that chronic hemodialysis maintenance therapy promotes gradual loss of inspiratory muscle strength, which deteriorates further with the duration of hemodialysis therapy [33]. Whether rehabilitation programs will prove to be beneficial for these patients' functional performance status is of interest and needs to be further investigated.

Finally, we noted that the CV % predicted reflecting small airway disease and maldistribution of ventilation [6, 7] and the *P*_*i*max⁡_ improved after hemodialysis. Both were correlated with the fluid depletion observed after the therapeutic session. This is beneficial for the patient's respiratory function. Whether aggressive hemodialysis treatment in preoperative patients can imply a better survival outcome after surgery needs also to be assessed.

## 5. Conclusions

We conclude that dyspnea is one of the most prominent symptoms in patients with CKD receiving hemodialysis maintenance therapy, which improves and often subsides after a therapeutic session. Dyspnea is correlated with the observed decrease in the neuromuscular drive as it is expressed by the mouth occlusion pressure (*P*_0.1_). The ensuing decreased neuromechanical dissociation can be attributed to (a) better neuromechanical muscle coupling, (b) the hypoventilation due to the alkalization of body fluids, (c) the increased inspiratory muscle strength after hemodialysis, and (d) the improved function of the small airways as it is expressed by the increased CV in the majority of the study patients, correlated with the weight loss after the hemodialysis. Further studies are needed to elucidate the impact of CKD and hemodialysis maintenance therapy on the patient's symptoms and respiratory function in order to improve patient's care.

## Figures and Tables

**Table 1:** Anthropometric and renal function data of the 25 patients before undergoing hemodialysis. Values are mean ± SD.

Subjects (*n*)	25
Age (yrs)	52.0 ± 11.0
Gender (M/F)	15/10
Height (cm)	161.0 ± 0.3
Weight (before hemodialysis) (Kg)	74.0 ± 12.0
Smokers/never smokers	13/12
Duration of dialysis (years)	4.7 ± 4.0
Hct (%)	37.0 ± 3.0
Urea (mg/dL)	141.0 ± 26.0
Creatinine (mg/dL)	10.0 ± 2.0
Potassium (mg/dL)	5.5 ± 0.5
Calcium adj (mg/dL)	10.0 ± 0.5

*n*: number of subjects before and after hemodialysis; M/F: male/female; calcium is adjusted to the albumin levels.

**Table 2:** Body weight, mMRC scale, SBN_2_ data (23 patients), and maximum static mouth pressure before and after hemodialysis.

	Before dialysis *n* = 25	After dialysis *n* = 25	*P* value
Body weight (Kg)	74.0 ± 12.0	71.0 ± 12.0	<0.001
BMI (Kg/m^2^)	26.0 ± 3.0	25.0 ± 4.0	<0.001
mMRC (acc)	2.0 ± 0.8	0.6 ± 0.7	<0.001
Δ*N*_2_/Δ*V*, % pred.	127.0 ± 25.0	86.0 ± 28.0	<0.001
CC % pred.	90.0 ± 28.0	77.0 ± 22.0	0.03
CV % pred.	125.0 ± 39.0	92.0 ± 41.0	<0.001
FRC % pred.	91 ± 27	88 ± 24	NS
*P*_*i*max⁡_ % pred.	70.0 ± 14.0	109.0 ± 15.0	<0.001
*P*_*e*max⁡_ % pred.	103.0 ± 24.0	109.0 ± 27.0	NS

Values are mean ± SD obtained in the seated position. Unless otherwise specified, values are expressed as % predicted. Statistical significance for mMRC tested with Wilcoxon's signed rank test before and after dialysis. Student's paired *t*-test is used for all the other lung function parameters. BMI: body mass index; mMRC: modified medical research council dyspnea scale; *n*: number of subjects; Δ*N*_2_/Δ*V*: slope of phase III; CC: closing capacity; CV: closing volume; FRC: functional residual capacity; *P*_*i*max⁡_: maximum static inspiratory pressure; *P*_*e*max⁡_: maximum static expiratory pressure.

**Table 3:** Pattern of breathing and arterial blood gases of the 25 ESKD patients before and after dialysis.

	Before dialysis *n* = 25	After dialysis *n* = 25	*P* value
*V*_*T*_ (lt)	0.7 ± 0.2	0.6 ± 0.2	NS
*T*_*E*_ (sec)	1.8 ± 0.5	1.7 ± 0.5	NS
*T*_*I*_ (sec)	1.5 ± 0.3	1.4 ± 0.3	NS
*V*_*T*_/*T*_*I*_ (lt/sec)	0.5 ± 0.2	0.4 ± 0.2	NS
*T*_*I*_/*T*_TOT_ (sec)	0.4 ± 0.04	0.5 ± 0.03	NS
*V*_*E*_ (lt/min)	13.0 ± 4.0	12.0 ± 5.0	NS
RR (breaths/min)	19.0 ± 5.0	20.0 ± 4.0	NS
*P*_0.1_ (cm H_2_O)	3.0 ± 0.3	0.7 ± 0.2	<0.001
*P*_0.1_/*V*_*T*_% VC pred., acc	0.2 ± 0.1	0.05 ± 0.02	<0.001
pH	7.4 ± 0.1	7.5 ± 0.1	<0.001
PaO_2_ (mm Hg)	98.0 ± 11.0	97.0 ± 14.0	NS
PaCO_2_ (mm Hg)	31.0 ± 4.0	33.0 ± 5.0	0.02
HCO_3_ (mmol)	20.0 ± 4.0	25.0 ± 3.0	<0.001

Values are mean ± SD obtained in the seated position. Unless otherwise specified, values are expressed as % predicted. Statistical significance tested with Student's paired *t*-test before and after dialysis. *V*_*T*_: tidal volume; *V*_*E*_: minute ventilation; *T*_*E*_: duration of expiration; *T*_*I*_: duration of inspiration; *T*_TOT_: total cycle duration; *V*_*T*_/*T*_*I*_: mean inspiratory flow; *T*_*I*_/*T*_TOT_: duty cycle; *f*: frequency of breathing; *P*_0.1_: mouth occlusion pressure; % pred. VC: vital capacity % predicted; PaO_2_: arterial pressure tension of oxygen; PaCO_2_: arterial pressure tension of carbon dioxide; HCO_3_: bicarbonate anion.

## Abbreviations

(Δ): Change
% pred.: Percent predicted
ARDS: Adult respiratory distress syndrome
ATS/ERS: American Thoracic Society/European Respiratory Society
BMI: Body mass index
CC: Closing capacity
CKD: Chronic kidney disease
CRF: Chronic renal failure
COPD: Chronic obstructive pulmonary disease
CSF: Cerebrospinal fluid
CV: Closing volume
CXR: Chest X-ray
DL_CO_: Diffusing capacity for carbon monoxide
ESKD: End stage kidney disease
FVC: Forced vital capacity
FRC: Functional residual capacity
IC: Intensive care unit
GFR: Glomerular filtration rate
KRT: Kidney replacement therapy
KOQI: Kidney outcome quality initiative
mMRC: Modified medical research council dyspnea scale
NECOSAD: Netherlands Cooperative Study on Adequacy of Dialysis
PaO_2_: Arterial oxygen tension
PaCO_2_: Arterial carbon dioxide tension
*P*_*e*max⁡_: Maximum static expiratory mouth pressure
*P*_*i*max⁡_: Maximum static inspiratory mouth pressure
*P*_0.1_: Pressure 0.1 second
*P*_0.1_/*V*_*T*_ % pred. VC ratio: The mouth occlusion pressure to tidal volume expressed as percent predicted of the vital capacity (VC) index.
RR: Respiratory rate
RV: Residual volume
SD: Standard deviation
SE: Standard error
SBN_2_: Single breath nitrogen test
TLC: Total lung capacity
*T*_*E*_: Duration of expiration
*T*_*I*_: Duration of inspiration
*T*_*I*_/*T*_TOT_: Duty cycle
*T*_TOT_: Total respiratory cycle duration
*V*_*E*_: Minute ventilation
*V*_*T*_: Tidal volume
*V*_*T*_/*T*_*I*_: Mean inspiratory flow
Wt: Weight
Δ*N*_2_/Δ*V*: Slope of phase III.
